# Scaling Up mHealth: Where Is the Evidence?

**DOI:** 10.1371/journal.pmed.1001382

**Published:** 2013-02-12

**Authors:** Mark Tomlinson, Mary Jane Rotheram-Borus, Leslie Swartz, Alexander C. Tsai

**Affiliations:** 1 Centre for Public Mental Health, Department of Psychology, Stellenbosch University, Stellenbosch, South Africa; 2 Semel Institute for Neuroscience and Human Behaviour, University of California at Los Angeles, Los Angeles, California, United States of America; 3 Centre for Public Mental Health, Department of Psychology, Stellenbosch University, Stellenbosch, South Africa; 4 Chester M. Pierce, MD Division of Global Psychiatry, Department of Psychiatry, Massachusetts General Hospital, Boston, Massachusetts, United States of America; 5 Center for Global Health, Massachusetts General Hospital, Boston, Massachusetts, United States of America

## Abstract

Mark Tomlinson and colleagues question whether there is sufficient evidence on implementation and effectiveness to match the wide enthusiasm for mHealth interventions, and propose a global strategy to determine needed evidence to support mHealth scale-up.

**Summary Points**

- Despite hundreds of mHealth pilot studies, there has been insufficient programmatic evidence to inform implementation and scale-up of mHealth.
- We discuss what constitutes appropriate research evidence to inform scale up.
- Potential innovative research designs such as multi-factorial strategies, randomized controlled trials, and data farming may provide this evidence base.
- We make a number of recommendations about evidence, interoperability, and the role of governments, private enterprise, and researchers in relation to the scale up of mHealth.

## What Is the Problem?

There are over 6 billion mobile phone subscribers and 75% of the world has access to a mobile phone [1]. Service and care providers, researchers, and national governments are excited at the opportunities mobile health has to offer in terms of improving access to health care, engagement and delivery, and health outcomes [2]. Interventions categorized under the rubric “mobile health” or “mHealth”—broadly defined as medical and public health practice supported by mobile devices [2]—span a variety of applications ranging from the use of mobile phones to improve point of service data collection [3], care delivery [4], and patient communication [5] to the use of alternative wireless devices for real-time medication monitoring and adherence support [6].

A recent World Bank report tracked more than 500 mHealth studies, and many donor agencies are lining up to support the “scaling up” of mHealth interventions [7]. Yet, after completion of these 500 pilot studies, we know almost nothing about the likely uptake, best strategies for engagement, efficacy, or effectiveness of these initiatives. Currently, mHealth interventions lack a foundation of basic evidence [8], let alone a foundation that would permit evidence-based scale up. For example, in Uganda in 2008 and 2009 approximately 23 of 36 mHealth initiatives did not move beyond the pilot phase [9]. The current enthusiasm notwithstanding, the scatter-shot approach to piloting mHealth projects in the absence of a concomitant programmatic implementation and evaluation strategy may dampen opportunities to truly capitalize on the technology. This article discusses a number of points pertinent to developing a more robust evidence base for the scale up of mHealth interventions. The issues raised are primarily conceptual and methodological.

Industry's increasing role in pushing for mHealth scale up is also a cause for concern. At a recent mHealth conference in South Africa, there were repeated calls for scale up of mHealth initiatives across low- and middle-income countries (LAMICs). Many of these calls emanated from industry representatives rather than researchers, governments, or care providers [10]. It is likely that private enterprise has a quite different understanding of what scale up means, with growing market share, rather than improved health outcomes, at the core of their mission. The growing involvement by industry, predominantly mobile phone providers, warrants some caution in addition to perhaps a code of practice. Public–private partnerships will be of central importance in the evolution of the mHealth field (as we discuss later), but this cannot happen at the expense of good science and good public health.

In some ways, mobile technology has a magical appeal for those interested in global public health over and above the advantages that have been proven with good evidence [11]. Part of this magical promise is that mobile technologies may solve one of the most difficult problems facing global health efforts—that of structural barriers to access. Travel, especially to remote areas in LAMICs, is expensive, destructive to the environment, time-consuming, and exhausting and physically challenging to many. In the global health field, there are many practitioners whose personal and working lives are substantially disrupted by travel of this nature. Mobile technology may hold out the promise of a world within which these difficulties can be minimised or eliminated. There is an obvious appeal for people from higher-income contexts being able to remain at home and in their offices while interacting with and improving the health of people very far away and in straitened circumstances. Mobile technology may hold out the promise that the visceral challenges of travel and complex intercultural contact, so much a feature of the global health enterprise, may now be a thing of the past [12].

## Current State of the Evidence

While enthusiasm for effective mHealth interventions in sub-Saharan Africa is high, little is known about their efficacy or effectiveness. Most randomized trials of mHealth interventions have employed text message reminder systems. Two systematic reviews have described a robust evidence base for the use of text message reminders to improve attendance at health care appointments [13],[14]. Yet, none of the studies included in these reviews was conducted in resource-limited settings. Similarly, few randomized trials evaluating the use of text message reminders to improve medication adherence for people with chronic illnesses have been conducted in LAMICs [15]–[18]. Three randomized trials studying HIV treatment adherence found benefits [19],[20] and one found no impact [21],[22]. Two recent systematic reviews [23],[24] found modest and suggestive evidence for the benefits of mHealth technology, and while both reviews recommended implementation, they argued that high quality (and adequately powered) clinical trials that measure clinical outcomes are essential.

The reviews of mHealth interventions would be more helpful if the results were organized according to 1) foundational functions (informing, training, monitoring, shaping, supporting, and linking to care); 2) content-specific targets (e.g., for Millennium Development Goal developmentally related tasks and challenges); and 3) local cultural adaptations (e.g., language) [25]. The inconsistency of results from mHealth studies demonstrates the importance of having an organizational framework.

## What Constitutes Evidence?

**Figure 1:**
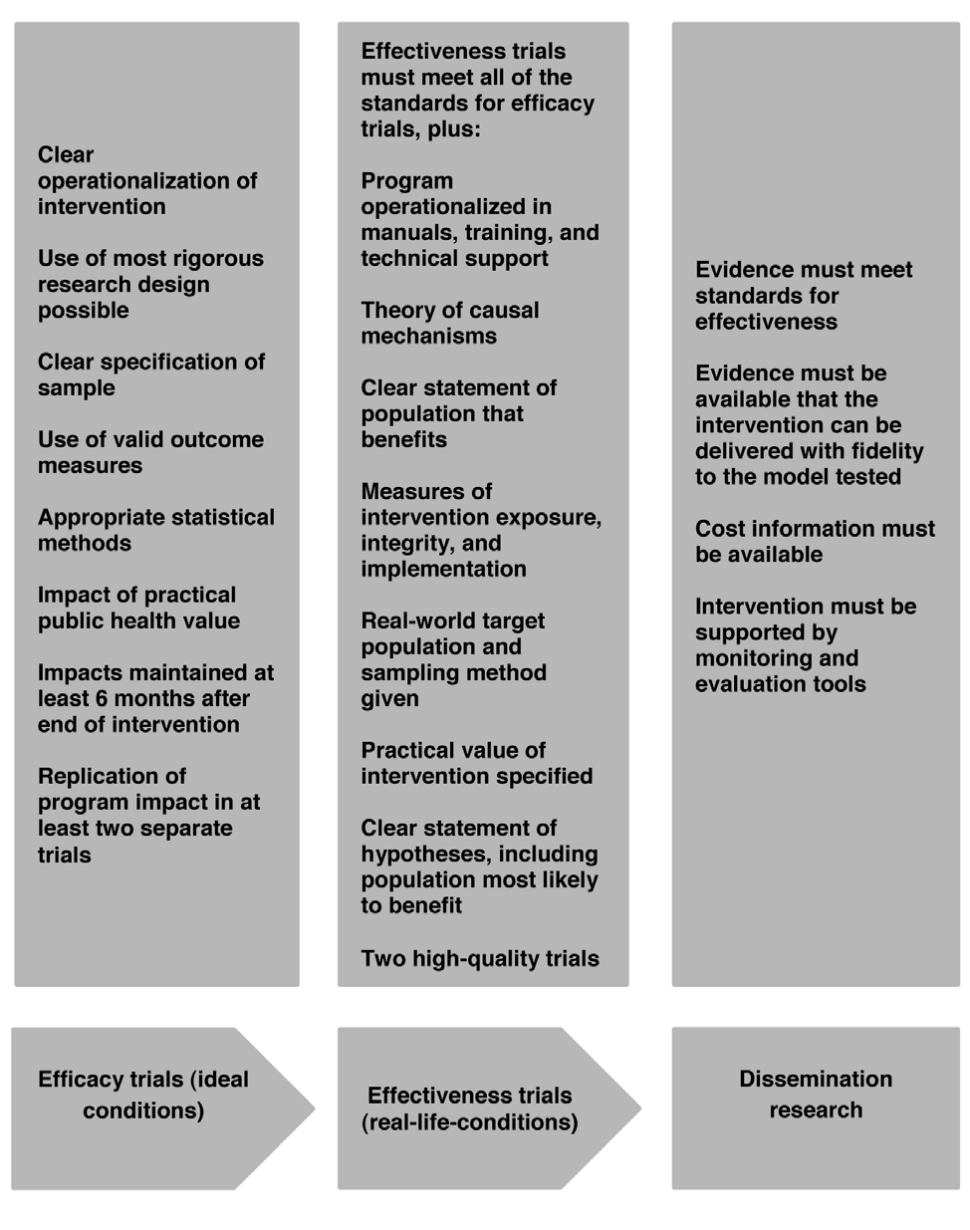
Research stages and standards. Adapted from Olds et al. [29] and Flay et al. [27].

The Institute of Medicine [26] and other communities of researchers [27],[28] have established standards for the phases of research that must be conducted in order to be considered efficacious, effective, and disseminated. Flay and colleagues [29] have adapted the evidentiary standards model published by the Society for Prevention Research [27]. These standards were developed in order to guide policy, research, and practice and provide a useful framework to determine what constitutes good and sufficient evidence. In this model (see Figure 1), scale up or country-wide implementation would be dependent on the completion (for each intervention) of (a) two high quality efficacy trials, (b) two high quality effectiveness trials, followed by (c) dissemination research that has established that the intervention can be delivered with fidelity to the model being tested, as well as (d) information about the intervention's costs. There are currently no mHealth interventions that meet these standards for scale up despite numerous calls to scale up mHealth projects.

Linked to the issue of standards for the phases of research is the question of theories of behaviour change. Aboud and Singla [30] have shown how programmes that simply provide health information (e.g., via SMS [short message service, or text messaging]) tend to be unsuccessful, while interventions providing skills through peer educators are more likely to be successful [30]. There are well validated theories of behaviour change common to many evidence-based interventions for prevention, diagnoses, and care, but none of the mhealth initiatives appear to be grounded in such theories [31]. We would argue that in the context of scarce resources, implementing untested mHealth interventions at scale without a theory of behaviour change is likely to result in many failed scale up projects and significant levels of wasted resources.

Finally, no major investments have been made to create a robust platform for mobile phones that could be used by designers of applications and electronic medical records that will allow cross-fertilization or integrated systems to be utilized [32]. Thousands of small applications have been propagated on closed-source platforms (e.g., iPhone applications and others) that each major mobile phone provider appears ready to replicate at high cost. Currently, a patient with two or more health conditions will have to make use of numerous applications for monitoring different health-related parameters such as medication adherence and health status, a disease-specific approach that he or she is unlikely to sustain [32]. Estrin and Sim make the case that there is a global communication network already in place to support an open mHealth architecture that could facilitate scalable and sustainable health information systems [32]. Interoperability will be critical to promote research initiatives. The largest investments to date in interoperable systems have been actively pursued by for-profit companies, given the staggering profits to be made in the proprietary applications market. What is needed is a concerted effort by governments, funders, and private enterprise to cooperate in order to set standards (e.g., number of bits) and to create a self-governing commercially viable ecosystem for innovation [32]. mHealth is in a period very similar to the early days of the Internet: not creating robust, interoperable platforms will ensure failure for mHealth initiatives to be scaled to improve health outcomes for at least the next decade.

## What Needs to Happen Next: From Black Box to High Utility

The current wave of mHealth interventions are the equivalent of black boxes. Each small entrepreneur or researcher includes whatever bells and whistles that their funding allows in an attempt to demonstrate efficacy. For example, hundreds of small pilot studies are finding whether text messaging works. Text messaging is more likely to work under a set of parameters:

- when there is follow-up;
- when the message is personally tailored;
- when the frequency, wording, and content are highly relevant.

Similar strategies are being experimented with for a range of topics, delivery strategies (web, phone, videos, social media sties), and populations. There are a set of principles that could potentially be established to identify the optimal strategies for delivering mHealth interventions. However, our current research is not aimed at identifying these principles and strategies. Each pilot study is examining whether their particular style of a black box application works better than not having any black box application. It is time to start funding randomized controlled trials of interventions that are based on researchers' best guesses about optimal implementation.

It is also time to consider the Multiphase Optimization Strategy (MOST) developed by Collins and colleagues [33]. The MOST strategy is grounded in an engineering approach and requires a two-stage process: 1) identifying the range of features that contribute to variation for a particular intervention; and 2) selecting a small set of factors and empirically testing them with a multi-factorial design. The initial set of factors to be screened might be determined on the basis of theory and/or experience and could be informed by research implementing evidence-based interventions with other delivery formats. The utility of such an approach has been demonstrated by Stretcher and colleagues [34] for web-based smoking cessation policies. Rather than having a single tested web-based, evidence-based intervention (EBI) that will then compete with other web-based EBI for smoking, there are a set of parameters that outline the optimal strategy for implementing a web-based programme. Similar strategies have long been adopted by health services researchers [35]. However, few of the existing studies utilizing mHealth delivery formats have adopted such an approach.

MOST is not the only approach that could potentially enhance the efficiency of existing mHealth studies. Duan [35] has advocated for the establishment of data farms. Nascent Internet companies such as Google, Yahoo, and Facebook provide informative case studies of data farms. Rather than use experimental research designs (such as randomized controlled trials), these companies can harvest data from billions of users of mobile, web, and social media, and computer-based interventions provide the evidence regarding the specific types of consumers who are attracted to specific types of delivery formats delivered with specific levels of doses at specific times. Data farms offer the opportunity to know the who, what, when, where, and how of reaching consumers [36]. Private enterprise has been outstanding at this function: mHealth needs to utilize their platforms and methods to optimize personal health.

Major donors could invest in creating a robust set of standards and a platform that can inform and support local adaptation of mHealth applications. The standardized features of the platform could then be available to all local technicians committed to improving the health of their local communities. At the very least, given that standards are expensive to establish, as well as often being complex and difficult to understand, one option is for an organization such as the World Health Organization to “certify” standards that meet particular criteria, or even to become a disseminator of standards. We also believe a global strategy for programmatic examination of the optimal features of the mobile platforms is needed, namely a platform that incorporates (for example) factorial designs to test the multiple features of interventions [37], the MOST strategy and even data farms. This could quickly identify and provide guidance to hundreds of thousands of programmers globally that could leverage donor investments to improve their communities' access to information, skills, telemedicine, or management of front line workers.

**Box 1. Recommendations for Scale Up of mHealth**

1. Existing standards for research should be reconsidered in order to provide guidance as to when scale up is appropriate.
2. mHealth interventions should be guided by a plausible theory of behaviour change and should use more than one technique depending on the targeted behaviour [38].
3. We need to establish an open mHealth architecture based on a robust platform with standards for app development which would facilitate scalable and sustainable health information systems.
4. Implementation strategies such as factorial designs that are able to test the multiple features of interventions must be explored, in order to provide the necessary evidence base.
5. Scale-up of mHealth in LAMICs should be preceded by efficacy and effectiveness trials so that they are founded on an appropriate evidence base.
6. Governments, funders, and industry must cooperate in order to set standards to create a self-governing commercially viable ecosystem for innovation.

## Abbreviations

EBI: evidence-based intervention
LAMIC: low- and middle-income country
mHealth: mobile health
MOST: Multiphase Optimization Strategy
